# Using graphic medicine in teaching multicultural nursing: a quasi-experimental study

**DOI:** 10.1186/s12909-023-04223-2

**Published:** 2023-04-17

**Authors:** Małgorzata Lesińska-Sawicka

**Affiliations:** Department of Nursing, State University of Applied Sciences in Piła, Piła, Poland

**Keywords:** Multicultural nursing, Graphic medicine, Comics, Teaching methods

## Abstract

**Background:**

Comics, as an art form that combines words and images, can be used with great success in teaching nursing students. Teaching content on multicultural issues is not easy, especially since, in addition to knowledge, it is important to pay attention to communication skills, attitudes of respect, openness or empathy, among others. It is difficult to recognize or discuss these attitudes without student involvement. Graphic stories, comic strips provide such opportunities and facilitate learning new content, even those difficult to communicate naturally and spontaneously. The aim of this paper is to present the possibilities of using the graphic method, in particular comics and graphic novels, in teaching nursing, based on the example of multicultural nursing.

**Methods:**

Quasi-experimental intervention study with a quantitative approach, guided by the STROBE tool The survey was conducted March–May 2022 among State University of Applied Sciences in Piła students. First, students’ knowledge of cultural issues was assessed, then they were randomly assigned to two groups. One group had classes using a comic book, and the other group had classes using traditional methods. After the class, the students’ knowledge was assessed again. Descriptive statistical analyses were performed to obtain the mean, median, standard deviation (SD). Data followed a normal distribution. Data was verified by the t-Students test for independent groups.

**Results:**

Respondents’ knowledge on cultural issues before taking the course was satisfactory, with a mean score of 19.1. After completing the course, knowledge on cultural issues increased and was rated as very good and the mean score for all respondents was 26.9. Statistical analysis showed that there was statistical significance between the groups and the number of points achieved in the posttest. Respondents in the intervention group scored higher than those in the comparsion group.

**Conclusion:**

The use of the graphic method, which is one of the forms of active learning, in teaching cultural content to nursing students has positive didactic effects. Students achieve better learning outcomes in terms of knowledge, skills and attitudes in a way that is more engaging. This makes learning difficult topics, such as a cultural issue, more effective.

It would be worth considering using this method for other courses and/or at other universities as well.

## Background

Modern nurses need more than merely manual skills. They must have the ability to assess their own knowledge and the competences related to practice and evidence-based research. They should cooperate in multidisciplinary teams and demonstrate critical thinking skills as well as the ability to make the correct decisions in difficult situations [35]. They should also take into consideration social and cultural skills while providing care to patients and their families and possess good communication skills.

A broad scope of skills and knowledge of nursing students are a challenge for nursing education leaders. These teachers are additionally confronted with a rapidly changing healthcare environment, increased demand for specialist education and the varied professional competences that are required in the profession of a nurse [15]. The increasingly higher requirements posed to the nursing staff also lead to changes in the teaching environment, and new educational methods are replacing the traditional lecture-based style with modern learning and teaching styles and strategies, which improve the involvement of students in interactions with other people, help them build a stronger rapport, develop soft skills and clinical skills, and promote self-learning to improve the critical thinking and decision-making skills among students [1]. The way in which students obtain and store information, and how they focus in class, influences the processes of knowledge acquisition, remembering, memorising, and consolidating information and using it in practice.

### Graphic medicine

The term graphic medicine was used for the first time by Williams, a British physician and author of comic books, to introduce an interdisciplinary method of communicating the subjective aspects of health problems. Later, other scientists and healthcare specialists joined Williams’s team, thus encouraging others to expand the ways of analysing illness, medicine, and disability, and to reconsider the scope of the notion of “health” [14]. In 2009, graphic medicine began to gain academic recognition, when Green started an optional course in *Graphic Storytelling and Medical Narratives* for fourth-year students at the w Penn State College of Medicine in Hershey, USA. In 2010, the first scientific conference dedicated to graphic medicine, entitled *Comics and Medicine: Medical Narrative in Graphic Novels*, was held at the London University.

One of the pioneers of graphic medicine was Czerwiec, who was also known as the *Comic Nurse.* She talks about herself as a nurse who uses comics to reflect on the complexity of illness and care [13]. Her “crayon revolution” theory has become a cornerstone for creating and using comics in healthcare. She claims that every person has an innate visual language, which may be accessed with use of such simple tools as crayons. This is why pictures that represent emotions, thoughts, and condition are more desirable than realistic presentations prepared with graphic pathography [27], i.e., narration about the illness in graphic form.

The stakeholders in graphic medicine may be the patients, their carers, and families, students of medical faculties, the healthcare staff, as well as literature, culture and graphic theoreticians. Graphic medicine enhances critical thinking in patients, and it prepares healthcare professionals and students to reach beyond the limiting framework of the analysis of facts and empirical data. Thus, it fits into the scope of narrative medicine, which strives to improve healthcare by providing valuable tools that are necessary to competently accept the testimonies of people about themselves, in order to recognise, acknowledge, and interpret the stories of others and allow them to move on to action [11]. Comics and graphic novels that draw from the principles of narrative allow patients and healthcare workers find a meaning in suffering, establish better relationships, identify all misconceptions that result from cultural prejudice or inaccurate information which might affect the diagnosis, treatment, and care [44].

### Comic book or graphic novel

The use of comic books in healthcare dates back to the 1860s. The first comics addressed public health issues. In the 1940s, the *True Comics* series began to appear. It was used to popularise scientific achievements in the field of medicine, encouraged people to choose medical sciences as their professional career and presented medicine as a humanitarian and noble endeavour [22]. However, after the establishment of the graphic medicine consortium in 2007, graphic narratives about experiences related to illness, such as Justin Green’s *Binky Brown Meets the Holy Virgin Mary* (1972), *The Spiral Cage* by Al Davison (1990), or the Pulitzer award winner *Maus* by Art Spiegelman (1991) as well as *Our Cancer* Year by Harvey Pekar and Joyce Brabner (1994), once more attracted the attention of scientists, physicians, teachers, and representatives of narrative and holistic medicine [27].

According to the subject literature, comics are currently successfully used, among others, in teaching students of nursing and medicine [2, 20, 21], in mitigating difficult emotional states in patients who were misdiagnosed [28], to show examples of good and bad practices and the ways to avoid correcting malpractice errors [27] as well as in consolidating knowledge about health [16].On the one hand, they are helpful for patients who want to learn more about their illness, while on the other hand, they provide healthcare workers with a new point of view on personal experiences related to illness (especially with respect to fears, which are often not mentioned by patients in the clinical environment) as well as misconceptions concerning the illness and its treatment, which might affect following the instructions and the prognosis [30].

One of the advantages of using comics instead of traditional printed materials is the fact that images affect the audience in a different, more emotional way. This emotional bond is reinforced by using an icon or symbol – any image used to represent a person, a place, a thing, or an idea. Such icon or symbol conveys both information and emotion, as it encourages the reader to identify with it. For example, a “smiley” face, thanks to its simplified form, appeals to nearly every human, regardless of their skin colour, ethnic origin, gender, etc., while a detailed photograph of a human face may not affect people from different backgrounds in the same way [20]. While reading a comic, the reader has to pay attention to two forms of information: visual and textual at the same time. Moreover, the illusion of “movement” in a comic book occurs only in the mind of the reader, who connects two static images when the eyes cross the space that divides two drawings.

Reading a comic requires interpreting not only the text, but also images, because the reader has to cope with two coding systems, which sometimes function independently, and, at other times, interact with each other. As a hybrid textual and pictorial format, the comic has “double narrative paths” [18], which require the reader to develop several strategies to assign meaning to various presented possibilities. Thus, a comic does not present a single, unquestionable message. Instead, the readers create their own interpretation creating a general meaning by referring the words and images in the comic to their own experiences. Every reader individualises his or her own reaction, which means that a comic is often characterised by the presence of multiple messages, where visual and linguistic codes coexist and interact. As a result, a comic does not have one “correct” or absolute meaning, but rather more or less important alternative interpretations [10]. At first sight, such “multiplicity of messages” may seem to be a disadvantage for a resource that is designed to present information about health, but, obviously, there is also high uncertainty related to the illness. Two patients with the same condition may experience different symptoms and react to treatment in different ways. It is precisely the ambiguity of a comic that may help capture this variety of experiences [34].

The aim of this paper is to present the possibilities of using the graphic method, in particular comics and graphic novels, in teaching nursing, based on the example of multicultural nursing. Subject literature demonstrates that, apart from educating patients, these materials are increasingly often used in teaching future healthcare professionals [26, 32, 39].

## Methods

### Study design

**Fig. 1:**
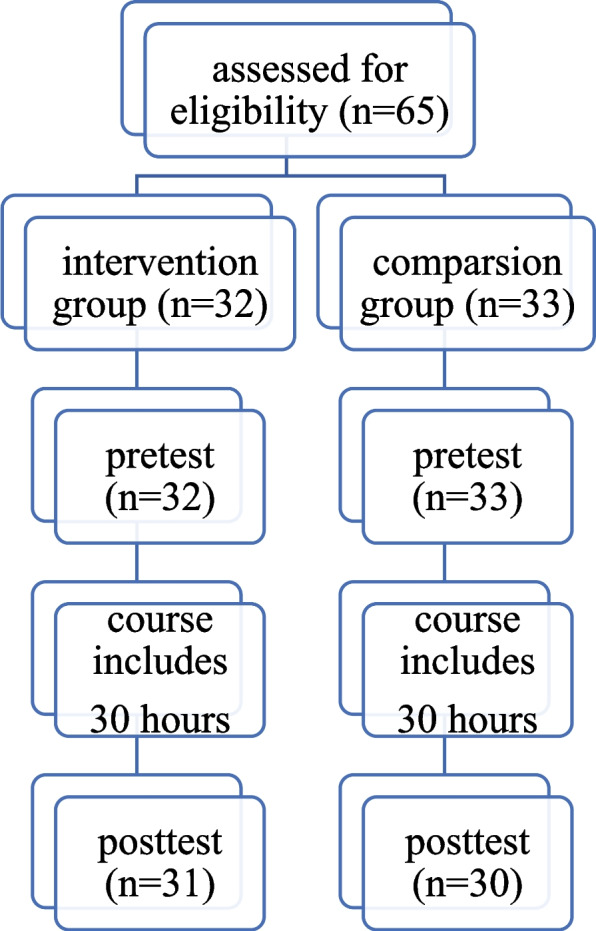
CONSORT diagram of quasi-experimental study with two groups

Quasi-experimental intervention study with a quantitative approach, guided by the STROBE tool (Fig. 1).

The study was conducted from March to May 2022 at the State University of Applied Sciences in Pila, Poland.

Prior to the start of the Multicultural Nursing course, students were asked by their yearbook supervisor to participate in the survey. Respondents were informed about the purpose and character of the research, how to answer and return it to the person designated, the possibility of withdrawing from the survey during the survey without any consequences.

Before taking the survey, respondents verbally agreed to participate in the study.

They were then given a pretest with questions on basic knowledge of multicultural issues. Respondents gave written answers at the university in 30–35 min. Respondents were randomly divided into two groups:intervention group—had classes using the graphic method, cartoonscomparsion group—attended classes conducted using traditional methods; discussion was based on case descriptions.

After completing the course, respondents wrote a posttest. Respondents gave written answers at the university in 30–35 min.

The following scale was used to assess knowledge on cultural issues in the pre- and posttest:

- 26—30 points very good knowledge
- 21—25 points good knowledge
- 16—20 points satisfactory knowledge
- 15 points and below insufficient knowledge.

**Fig. 2:**
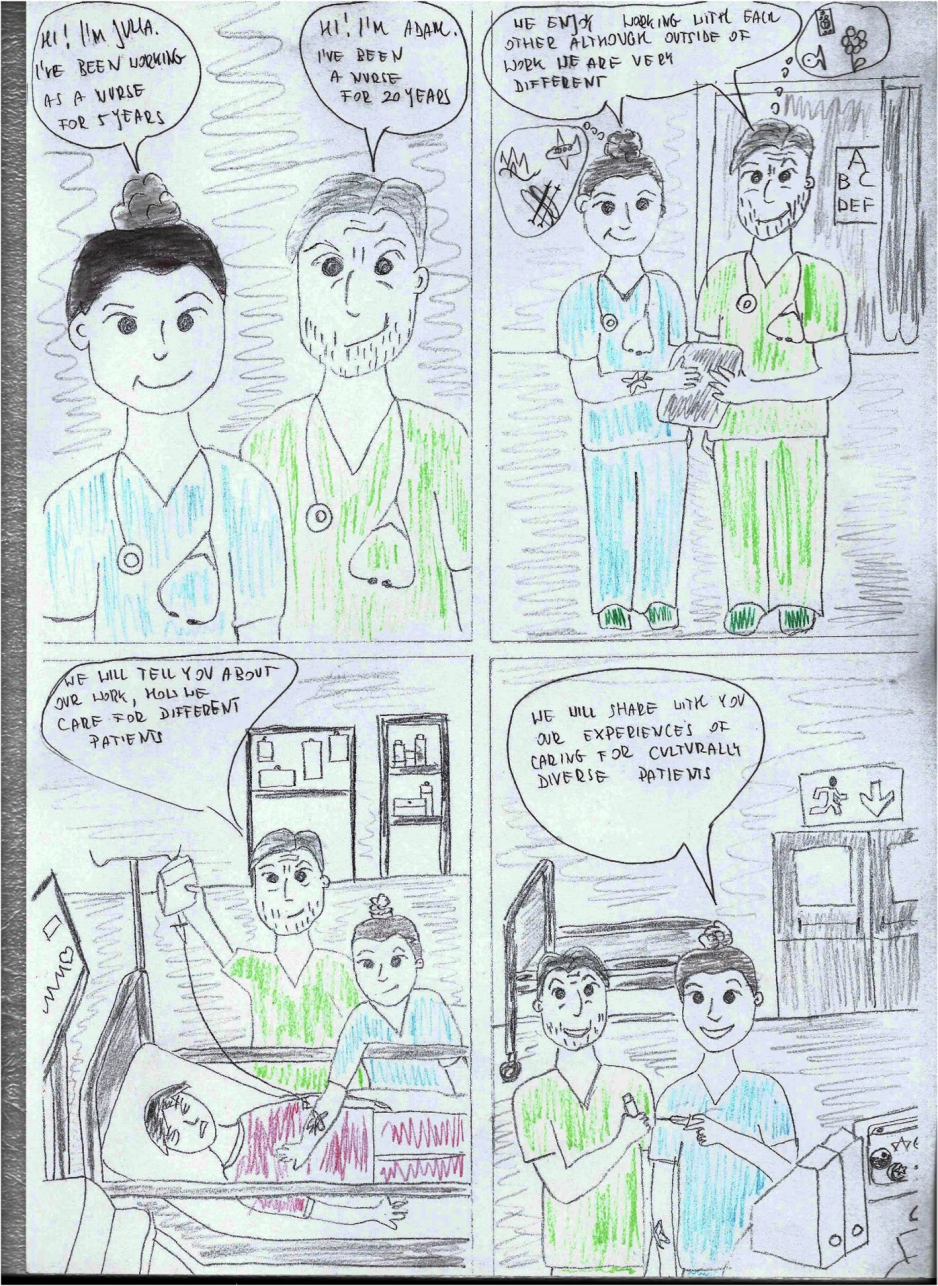
Presentation of the protagonists

**Fig. 3:**
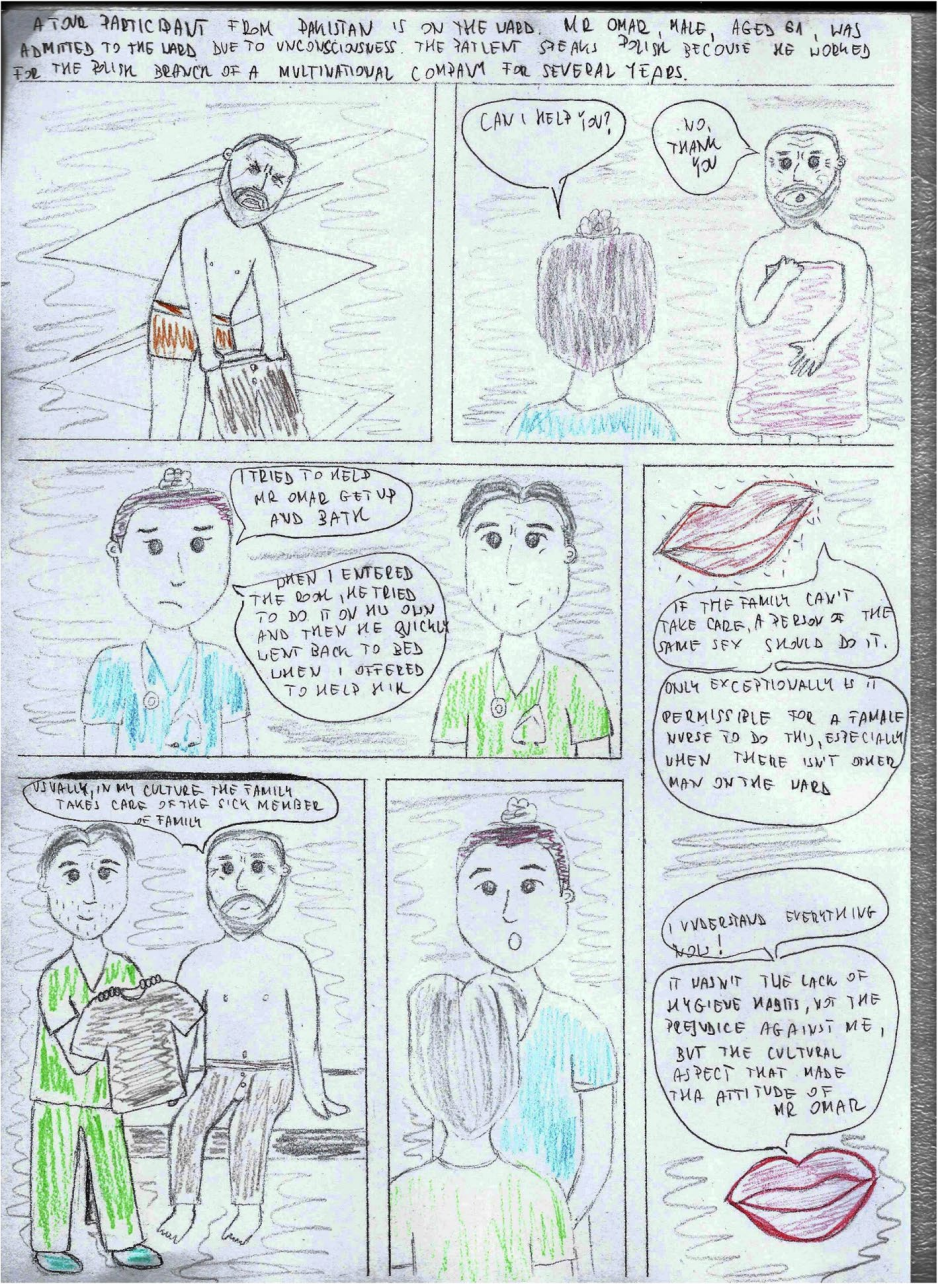
Gender and culture

**Fig. 4:**
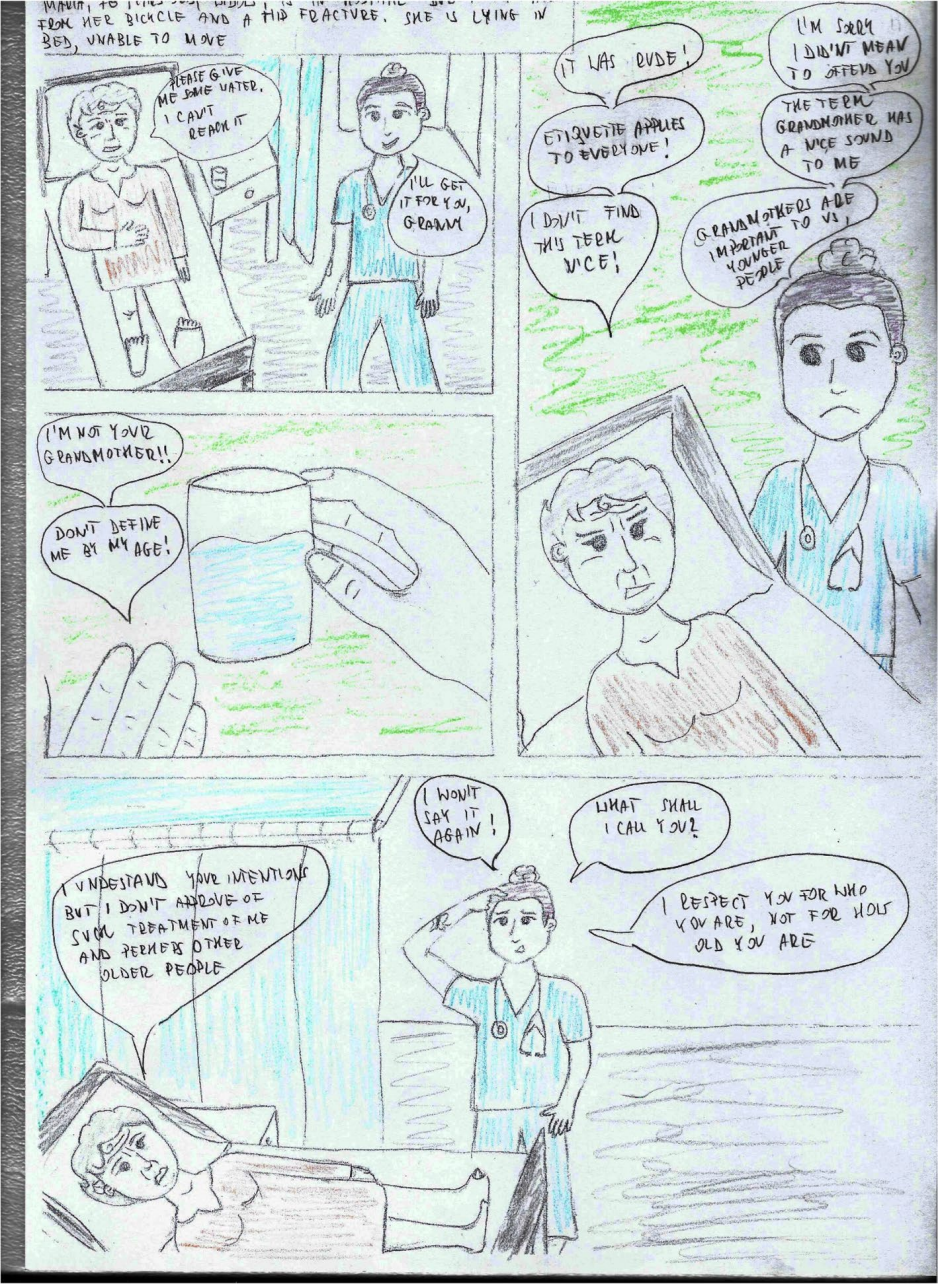
Age and culture

**Fig. 5:**
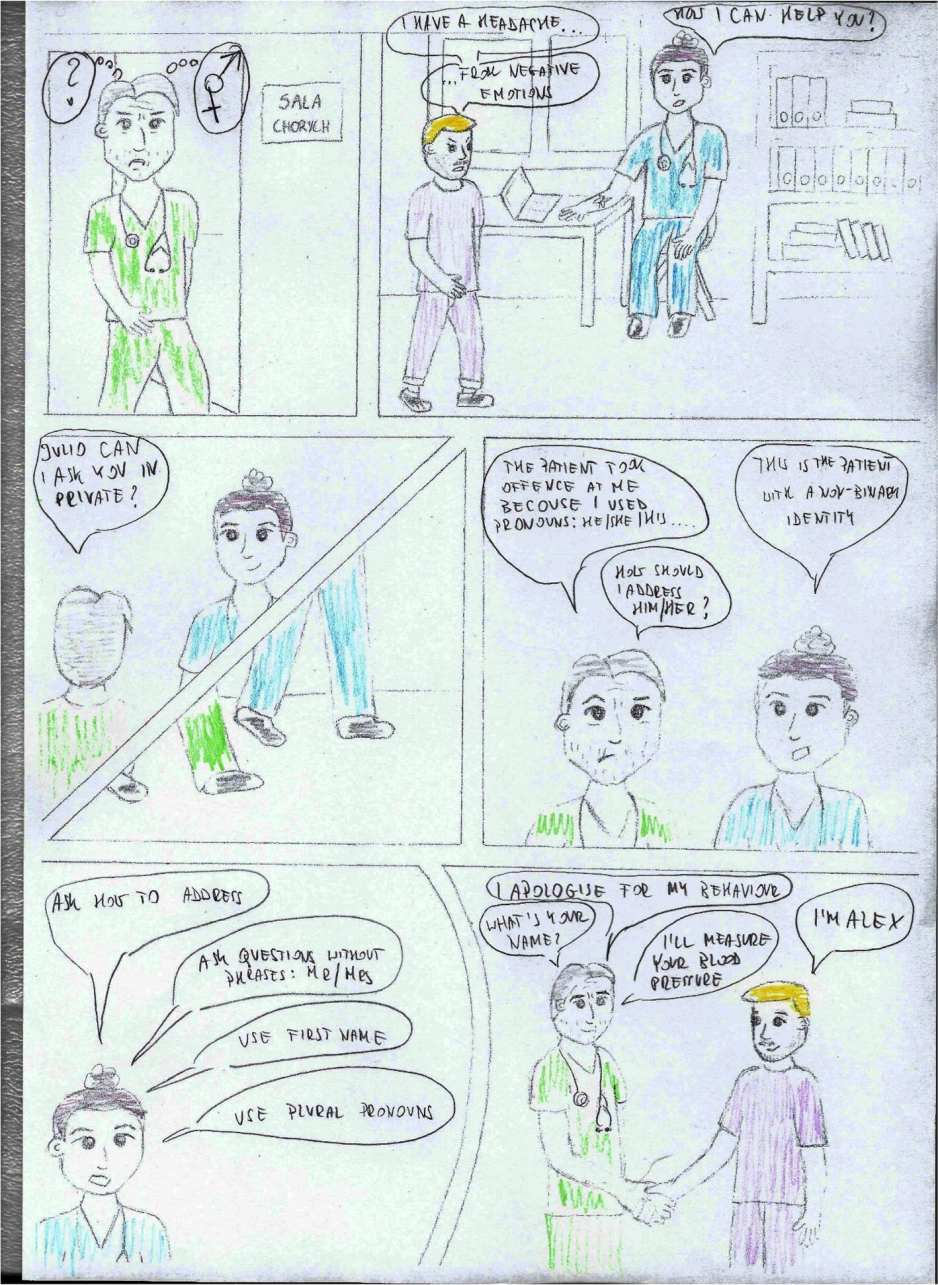
Nursing care of a non-binary patient

The course lasted 10 weeks, with one meeting per week for 3 lessons of 45 min each. The course covered issues such as gender, social structure, time, geography in culture, but also the impact of immigrant experiences, worldview, religion on the relationship between the nursing staff and the patient, etc. During the classes, both groups received a case report with the introduction of questions orienting them about and outlining the situational context (Table 1) and questions after reading the case studies concerning the situation, the problem, the presented ways of solving the problem, opinions on the attitudes of the participants in the situation, as well as suggestions for other solutions to the presented problem (Table 2). The difference in conducting case studies was that in the intervention group, students worked on a comic book telling the story of two nurses, Julia and Adam, who meet various patients in their work, e.g., a Pakistani, a Mexican, a neglected patient, a refugee from war zones, a student Italian, a young mother who does not want to vaccinate her child, etc. (Figs. 2, 3, 4, and 5). In turn, in the comparsion group, the same stories were presented in a descriptive form (Table 3).

**Table 1:** Proposed pre-briefing questions

Topic of the scene	Pre-briefing questions
Gender and culture	What is cultural gender, social gender, cultural-social gender?
	What are gender norms?
	Can cultural gender influence nursing care?
Age and culture	Does the age of a patient influence the manner of communicating with them?
	What is a stereotype and why do we use it?
	Have you ever been in a situation when you were treated based only on your age, not on your competences? How did you feel?
Nursing care of a non-binary patient	What does the abbreviation LGBTQ + mean?
	Who is a non-binary person?
	Do non-binary persons have different problems than binary persons?

**Table 2:** Proposed debriefing questions

Topic of the scene	debriefing questions
Gender and culture	What would you do in a similar situation?
	Why didn’t Mr Omar want to accept help from Julia?
	Are there any similarities and differences between the images of men and women in different cultures: European, American, African, Christian, Muslim, etc.?
Age and culture	Why was Mary so outraged by Julia’s words?
	Why did Julia address Mary in this way?
	In what other way could Julia address Mary?
	What is cultural humility?
Nursing care of a non-binary patient	What was Adam’s problem in communicating with the patient?
	What advice did Julia give Adam? Can you add anything else to that?
	What would you do in a similar situation?

**Table 3:** Example of a case study to be used by students

Ms Maria Dolores, a 45-year-old Mexican woman, has been living in Poland for a short time. She moved here with her family: her husband of Polish origin and two teenage kids. She has been hospitalised for two days due to heart problems
- Good morning María Dolores, buenos días, says Adam entering the room
- Good morning, the patient answers
- Maria Dolores, please put on your bathrobe. You are going to have a cardiac echo. During your doctor’s visit you gave consent to the examination. I will take you there, says Adam slowly
- Dónde? What is it? What are they going to harán? Va a ser un bole? The patient asks the questions loudly, gesturing wildly
- Don’t be afraid, the test does not hurt. The doctor will place the ultrasound head on your chest and will assess the condition of your heart, the valves, the blood flow, and so on. He will check if your heart is healthy
- No understand, no entiendo
- An ultrasound of you heart. Adam points to his chest
- Heart, my heart, ultrasound! Maria Dolores laments
- Yes, the doctor will have a look at your heart
- My heart, corazón, mi corazón. Tengo miedo. Es seguro? No moriré? The patient talks loudly
Julia arrives
- Maria Dolores, what’s going on? Why are you talking so loudly?
Julia turns to Adam:
- What happened?
- We cannot understand each other. Maria Dolores does not understand what I am saying, and I don’t understand her. I can see that she is agitated, Adam says
- Why don’t you try using a translator? Julia suggests
- Translator, traductor. The patient nods her head
Adam takes out his phone, and so does Maria Dolores
- It’s good I remembered to charge my phone, Adam says

### Setting

The respondents were nursing staff working in health care facilities in northern Greater Poland. Those who took part in the study were also first-year students of the second degree program at State Univerisity of Applied Sciences in Piła (ANS). The second degree program in the first year at ANS includes, as a compulsory course, a multicultural nursing course. The respondents were invited to participate in the study by the supervisors of each year group, who were not the authors of this paper. No incentive for participation was given. A total of 65 people agreed to participate in the study. Due to the absence of 4 people from the post-test, the final sample consisted of 61 students, with an overrepresentation of women (*n* = 59; 96.72%) compared to men (*n* = 2; 3.28%), which is similar to the proportion of the general population of nursing staff in Poland. Respondents were aged between 23 and 49 (M = 31.76, SD = 3.40) and worked in hospital wards (*n* = 50; 81.97%) and primary care (*n* = 11; 18.03%).

### Data analysis

The collected data were initially structured in an Excel spreadsheet and exported to the Statistica software (version 10.0) to perform the analysis. Descriptive statistical analyses were performed to obtain the mean, median, standard deviation (SD). Data followed a normal distribution. Data was verified by the t-Students test for independent groups.

### Ethical considerations

Before conducting the research, the necessary consent of The Committee on the Ethics of Scientific Research of the State University of Applied Sciences in Piła was obtained. Respondents were informed that they could withdraw from the study without giving any reasons and without any consequences, that their responses would be anonymised by removing any personal information and would be analysed with other responses to obtain aggregate results, no identifying information would be included in this dataset, there was no direct personal benefit associated with participation in this study.

## Results

**Fig. 6:**
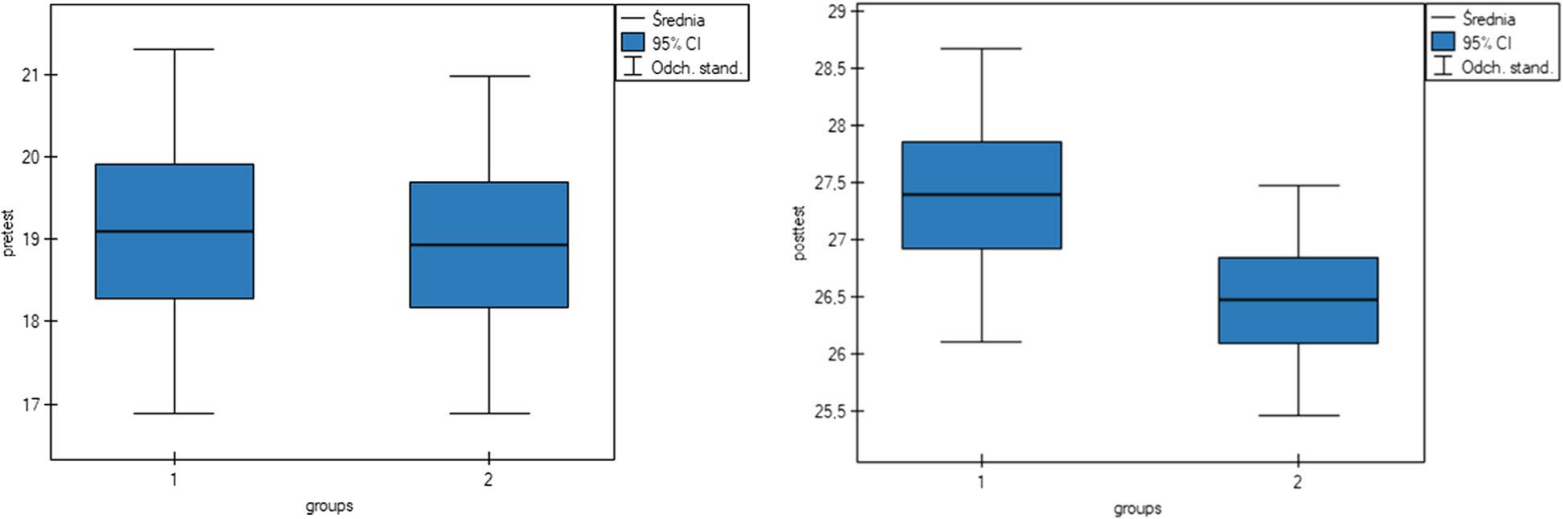
Average scores in the intervention (1) and comparsion (2) groups

A total of 61 students participated in the study, with 31 in the intervention group and 30 in the comparsion group. Respondents’ knowledge on cultural issues before taking the course was satisfactory, with a mean score of 19.1 and SD ± 2.58. After completing the course, knowledge on cultural issues increased and was rated as very good and the mean score for all respondents was 26.93 with SD ± 1.23. Statistical analysis showed that there was statistical significance between the groups and the number of points achieved in the posttest (*p:* 0.002) (Table 4). Respondents in the intervention group scored higher (mean score of 27.38) than those in the comparsion group (mean score of 26.46) (Fig. 6).

**Table 4:** Comparison of pretest and posttest results of the intervention and comparsion groups

Indicators	Intervention group *N* = 31			Comparsion group *N* = 30			t test/*p*
	Mean	SD	Median	Mean	SD	Median	
Pretest	19.06	2.21	18.28	18.93	2.04	18.16	0.29 *P:* 0.76
Posttest	27.38	1.28	26.91	26.46	1.00	26.09	3.10 *P:* 0.002

## Discussion

Active teaching methods are a way of working with groups which enable active learning through experience and active participation. They include a wide range of learning activities, such as critical pathways, problem-based learning, patient simulation, case-based learning, and mentors, interactive simulations, games, small group discussions, videos, blended learning, role-playing, pair share, flipped classroom, mini research projects, formative evaluations, quizzes and hands-on activities. They require from the learner understanding knowledge derived from the learning experience, while improving his or her learning and memory skills [19]. Active teaching strategies were introduced into teaching almost 40 years ago, but reports on their use in medical education are quite limited, despite a number of evident benefits they offer [7]. Active engagement makes the classes more interesting for the students, and makes them more willing to participate in tasks and work towards specific goals. The literature on the subject shows that educational methods that encourage students to think in a creative way, involve them in activities, and require that they arrive at a solution on their own are methods that also help to improve the safety and effectiveness of nursing intervention in patient care [35]. Active methods put the student at the centre of the learning process, foster critical thinking, improve decision-making skills [17], adaptability and communication skills [7] as well as increase learning efficiency resulting in better final grades and higher student satisfaction [1].

The graphic method used in this study is also a type of an active method. By using drawings when presenting new content and encouraging students to read speech bubbles in the cartoons, better learning outcomes were obtained compared to conventional teaching methods. Similar conclusions have been drawn by Afrasiabifar and Asadolah [1], Boctor [5], Logan et al. [31] and Romanowski et al. [38] who demonstrated that the use of active teaching methods increases learning efficiency, student satisfaction, increases students’ concentration during classes and assimilation of new content. At the same time, conflicting reports can be found in the literature on the subject. After studying 536 nurse educators, Bristol et al. did not identify any correlation between the use of active learning methods and National Council Licensure Examination pass rates [6].

The literature indicates the use of graphic stories in teaching nursing students [2, 20]. However, the information deals with general issues. The present study demonstrates the specific and purposeful use of cartoons in preparation for the nursing profession.

The cultural diversity of societies poses challenges for communities that accept migrants and immigrants and leads to the need to ensure culturally safe healthcare. Higher education institutions are responsible, among others, for building a fair society by including culture-related content in the teaching curricula in nursing [4]. Providing equitable, high quality nursing care, that will take into account the cultural background of the patient, is a legal and moral duty of every nurse [33]. A vital element of eliminating the health-based differences among patients is the inclusion of a deeper understanding of the multicultural context, in which the care is provided [24, 37]. Hence, cultural competences are an essential skill for nurses [44], who understand the fears of culturally different patients and demonstrate the sensitivity and knowledge of the risk factors that occur in different cultures.

Nurses should be competent in caring for patients, families, and groups from various cultural backgrounds [12], because when nurses provide culturally sensitive care, their clients are often more satisfied with the care [42]. As a result, nurses have to possess the cultural awareness of the individual needs of their clients and to adapt their practice in order to ensure safe and culturally equal care for everyone [3, 29]. However, nurses may not have the knowledge, skills, and attitudes that are necessary to ensure equitable care for everyone, including those from different cultural backgrounds [25]. Including the appropriate training into the curriculum, both during graduate and postgraduate studies, may help prepare a nurse, who has the cultural competences to provide culturally sensitive care, to identify the potential barriers in providing such care and to find efficient ways of avoiding or overcoming them [43]. The socially construed differences that exist between the nurse and the patient, and are based on cultural, racial, or ethnic identity, may be eliminated by improving the nurses’ knowledge about other cultures [9, 36, 40, 41]. Nursing education appears to be a perfect tool, that will enable cultural competences to be developed as part of the current and future nursing practice, as it plays the main role in the development of the skills, knowledge, and attitudes of nurses in providing individual, appropriate care to their patients [8]. In order to achieve the educational objectives, it is important to prepare an appropriate curriculum to define the strategies and styles of teaching.

Working with graphic novels is an interesting way to make the classes more attractive, but, apart from that, thanks to the simplified narrative and visual language, it enables students to acquire knowledge more easily, to analyse and understand the plot more quickly, and to develop the ability to draw conclusions, to empathise and to work in groups.

Cultural content is not easy to convey, especially when the aim is to emphasise sensitivity, humility, openness, respect, etc. It is difficult to recognise or discuss these attitudes without the active participation of the student. Graphic stories or comics offer such possibilities and, apart from that, they facilitate learning new content in a natural and spontaneous manner. According to Jacobs, comic books require their audiences to use so-called “multimodality”. This means that readers have to process various elements ‒ visual, spatial, and textual ones in the content ‒and then integrate them, in order to understand the meaning of the whole story. Neuroscientists have also demonstrated that looking at images and illustrations helps acquire knowledge more quickly and practices memory skills. Due to that, comics are very often used in textbooks and educational publications [23].

Graphic medicine, as a discipline operating both in the areas of comic books and healthcare, may help teachers and students analyse difficult issues related to medicine and health sciences, share their experiences and observations, facilitate the understanding of their own attitudes and the sources of the decisions made. A comic, as a form of art that combines words and images to tell a sequential story, allows us to reconceptualise our own experiences. 

### Limitation

Despite the value of the study, it also has some limitations. One limitation was that the survey was conducted with nursing students from a single university. The small number of respondents is also related to this. In addition, conducting the study among students despite the fact that the principal investigator had no direct contact with the study candidates could have created pressure among the students.

## Conclusion

The use of the graphic method, which is one of the forms of active learning, in teaching cultural content to nursing students has positive didactic effects. Students achieve better learning outcomes in terms of knowledge, skills and attitudes in a way that is more engaging. This makes learning difficult topics, such as a cultural issue, more effective.

It would be worth considering using this method for other courses and/or at other universities as well.

## Data Availability

The data analyzed in the study are available upon request to the first author.
